# Supplementary material for: Metabolomic Analysis of Actinic Keratosis and SCC Suggests a Grade-Independent Model of Squamous Cancerization
Source: Cancers (Basel). 2021 Nov 5;13(21):5560. doi: 10.3390/cancers13215560 (PMC8582912; doi:10.3390/cancers13215560)
Supplement: Supplementary file 1 [file cancers-13-05560-s001.zip › cancers-1425017-supplementary.pdf]

Supplementary Materials

Metabolomic Analysis of Actinic Keratosis and SCC Suggests a Grade-Independent Model of Squamous Cancerization

Valeria Righi [1,†], Camilla Reggiani [2,3,†], Elisabetta Tarentini [2], Adele Mucci [4], Alessia Paganelli [2,3], Anna Maria Cesinaro [5], Ema Mataca [5], Shaniko Kaleci [2], Barbara Ferrari [2], Marco Meleti [6] and Cristina Magnoni [2,*]

More than 40 metabolites in the tissues were identified; all the assignments are listed in Table S1. In the table are reported all the signals coming from mepivacaine, a local anesthetic used during surgery.

Table S1. List of ^{1}H and ^{13}C chemical Shift (δ, ppm) of metabolites. [a],^{1}H chemical shifts refer to Ala doublet at 1.48 ppm; [b],^{13}C chemical shifts refer to Ala at 19.3 ppm; [c], contributes to the 3.77, 57.1 ppm cross-peak.

	Metabolites	$\delta^{1}H^{a}$	$\delta^{13}C^{b}$	Assignment
**1**	Fatty acids	0.89	16.5	CH_3
		1.32–1.29	25.4, 32.6–34.2	$(CH_2)_n$
		1.59	27.5	CH_2-CH_2-C=O
		2.04	29.6	CH_2CH=CH
		2.27	36.1	CH_2-C=O
		2.78	27.9	=CH-CH_2-CH=
		5.32	128–130	-CH=CH-
**2**	Lactate	1.33 (d)	22.7	CH_3
		4.12 (q)	71.3	CH
**3**	Alanine	1.48 (d)	19.3	CH_3
		3.78	c	α CH
**4**	Valine	0.98 (d)	21.7	γ–CH_3
		1.04 (d)	20.7	γ–CH_3
		2.27	30.5	β–CH

		3.61	c	α-CH
**5**	Leucine	0.96	24.9	δ-CH_3
		0.97	25.3	δ–CH_3
		1.70	42.4	β–CH_2
		1.73	28.8	γ–CH_2
		3.72	c	α–CH
**6**	Lysine	1.47	22.6	γ–CH_2
		1.73	27.3	δ–CH_2
		1.88	32.9	β–CH_2
		3.03	39,7	ε–CH_2
		3.73	c	α–CH
**7**	Isoleucine	1.00 (d)	15.5	γ–CH_3
		0.95–0.93 (t)	11.7	δ–CH_3
**8**	Acetate	1.92 (s)	26.6	CH_3
**9**	Arginine	1.72,1.67		γ–CH_2
		1.91		β–CH_2
		3.25		δ–CH_2
		3.76	c	α-CH
**10**	N-acetyl	2.06 (s)	24.8	CH_3
**11**	Glutamine	2.13	29.0	β–CH_2
		2.45	33.4	γ–CH_2 (td)
		3.76	c	α–CH
**12**	Methionine	2.13 (s)	17.1	SCH_3
		2.62 (t)		γ-CH_2
		2.16		β-CH_2

		3.87		α-CH
**13**	Glutamate	2.36	36.4	γ–CH_2
		2.13,2.07	29.2	β–CH_2
		3.73	c	α–CH
**14**	Proline	3.43, 3.34		δ-CH_2
		2.01		γ-CH_2
		2.34, 2.08		β-CH_2
		4.12		α-CH
**15**	Threonine	1.32	21.8	γ–CH_3
		3.56	69	β–CH
		4.25	66.9	α–CH
**16**	α-glucose	5.24 (d)	92.6	1 CH
		3.55	72	2 CH
		3.72	73.1	3 CH
		3.42	76.4	4 CH
		3.82	71.9	6 CH_2
**17**	β-glucose	4.64 (d)	96.4	1 CH
		3.25	74.7	2 CH
		3.49	76.3	3 CH
		3.4	70.2	4 CH
		3.47	76.4	5 CH
		3.91	61.3	6 CH_2
**18**	Ribose	5.80		
**19**	Creatine	3.03 (s)	39.6	CH_3
		3.95 (s)	56.4	CH_2
**20**	Creatinine	3.05 (s)		CH_3

		4.06 (s)		CH_2
**21**	Ethanolamine	3.15 (t)		CH_2
		3.82 (t)		CH_2
**22**	Histidine	3.19		β-CH_2
		3.97		α-CH
		7.05 (s)		2-CH
		7.78 (s)		4-CH
**23**	Phenylalanine	3.28, 3.11		β-CH_2
		3.99		α-CH
		7.34	130.1	H-*ortho*
		7.37		H-*para*
		7.43	129.6	H-*meta*
**24**	Choline	3.20	56.6	$N(CH_3)_3$
		3.50	68.2	NCH_2
		4.08	56.5	OCH_2
**25**	Glycine	3.56 (s)	44.3	CH_2
**26**	Taurine	3.42 (t)	38.2	N-CH_2
		3.26 (t)	50.2	S-CH_2
**27**	Tyrosine	6.88		5 CH
		7.18		6 CH
**28**	Succinate	2.37 (s)	34.2	CH_2
**29**	Glycerol bound	4.30, 4.10	64.7	CH_2
		5.22	71.7	CH
**30**	Free Glycerol	3.56-3.65	63.3	1-CH_2
		3.81	72.7	2-CH
**31**	Glyceryl phosphorylcholine	4.32	60	α'-CH_2
		3.67	66.6	γ–CH_2
		3.24 (s)	56.5	N-CH_3

**32**	Phosphorylcholine	3.22	54.7	$N(CH_3)_3$
		3.61	67.3	NCH_2
	Aspartate	4.22	59.0	OCH_2
**33**		2.68–2.82	37.2	β-CH_2
		3.90		α-CH
**34**	Asparagine	2.85-2.96	26.3	β-CH_2
		4.01		α-CH
**35**	Pyruvate	2.39 (s)	29.1	CH_3
**36**	Pyroglutamic acid	2.03, 2.51	27.9	2,4 CH_2
		2.41	32.5	3 CH_2
		4.19	61.2	1 CH
**37**	Myo-inositol	3.53	72	1,3 CH
		4.06	71.3	2 CH
		3.63	73.1	4,6 CH
		3.27	74.4	5 CH
**38**	Scyllo-inositol	3.36 (s)	73.6	CH
**39**	Serine	3.87	59.2	α–CH
		3.94	63.2	β–CH_2
**40**	EtOH	1.18	17.5	CH_3
		3.65	56.7	CH_2
**41**	Glutathione	2.55		5 CH_2
		2.95		4 CH_2
		4.57		1 CH
**42**	Ascorbate	4.52		4 CH

		4.01	5-CH
**43**	Formate	8.48	$HCOO^-$
**44**	Mepivacaine	2.18 (bs)	CH_3
		2.23 (bs)	CH_3
		7.21 (d)	3,5-CH
		7.25 (t)	4-CH
**45**	Fumarate		

Table S2. *p values* derived from the comparison between healthy and AKI, healthy and AKII, healthy and AKIII and finally healthy and SCC.

Metabolites	Healthy vs :			
	P value AKI	P value AKII	P value AKIII	P value SCC
Glc	0,3245	0,6092	0,0662	0,1344
GSH	0,0359	0,1543	0,0542	0,1664
Asc	0,0021	0,0000	0,0034	0,0913
Lac	0,0002	0,0005	0,0007	0,0077
Ser	0,0652	0,2487	0,0220	0,0094
Gly	0,0715	0,0739	0,0011	0,0416
Myo	0,2300	0,5725	0,0746	0,2561
Tau1	0,0075	0,0036	0,0245	0,0127
Scy	0,1675	0,1946	0,1142	0,2681
Tau2	0,0111	0,0020	0,0962	0,0348
GPC	0,0882	0,1198	0,0110	0,0221
PC	0,0181	0,0094	0,0111	0,0886
Cho	0,3953	0,6824	0,2268	0,0281
EtA	0,0205	0,0574	0,0015	0,0019
Cr	0,3076	0,1712	0,0016	0,1501
Gln	0,0013	0,0006	0,0001	0,0012
PGA	0,2173	0,9849	0,0204	0,0171
Glu	0,0026	0,0004	0,0010	0,0015
Met	0,0010	0,0159	0,0158	0,0153
Nacetyl		0,6424	0,9933	0,4908
Ac	0,6520	0,6412	0,0378	0,2958
Ala	0,1195	0,0956	0,0232	0,0206
Val	0,0315	0,0530	0,0022	0,0305

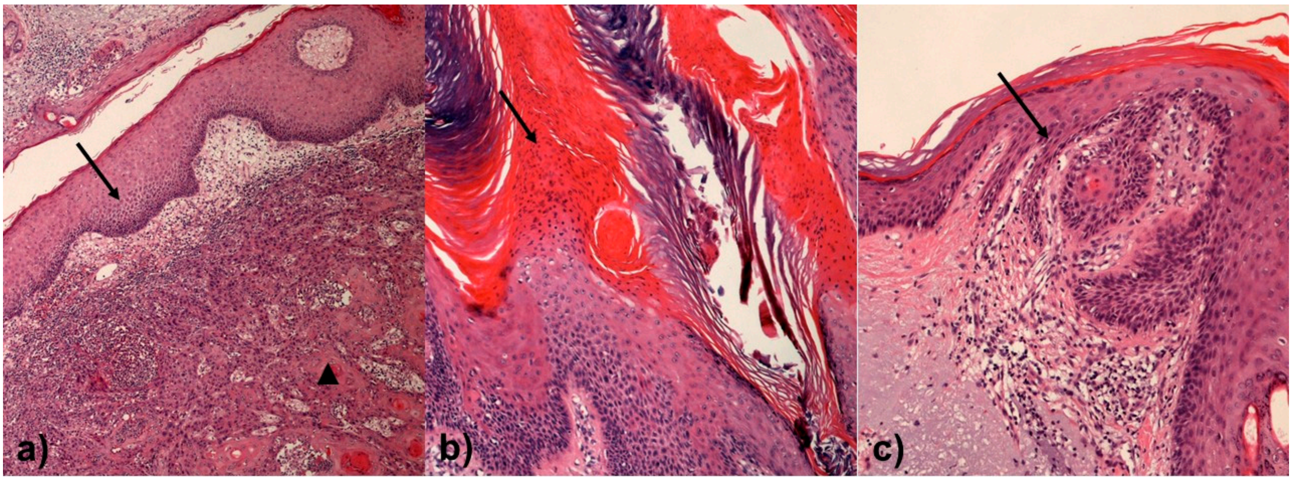

Figure S1. Selected histopathological section (hematoxylin and eosin, ×100 (A) and ×40 (B,C) magnification) corresponding to: (A) moderately differentiated SCC with Breslow thickness of 5 mm (triangle), associated with AK I (arrow); (B) AK III showing parakeratosis (arrow); (C) AK I characterized by the presence of hypertrophy (arrow).
